# A Multiplex RT-PCR Assay for *S. aureus, L. monocytogenes*, and *Salmonella* spp. Detection in Raw Milk with Pre-enrichment

**DOI:** 10.3389/fmicb.2017.00989

**Published:** 2017-05-31

**Authors:** Tian Ding, Yuanjie Suo, Zhaohuan Zhang, Donghong Liu, Xingqian Ye, Shiguo Chen, Yong Zhao

**Affiliations:** ^1^Department of Food Science and Nutrition, Zhejiang Key Laboratory for Agro-Food Processing, Zhejiang UniversityHangzhou, China; ^2^College of Food Science and Technology, Shanghai Ocean UniversityShanghai, China

**Keywords:** multiplex real-time PCR, enrichment step, *S. aureus*, *L. monocytogenes*, *Salmonella* spp., dairy farm environment, raw milk

## Abstract

This study firstly developed a multiplex real-time PCR (RT-PCR) technique combined with a pre-enrichment step to simultaneously detect *Staphylococcus aureus* (*S. aureus*), *Listeria monocytogenes* (*L. monocytogenes*) and *Salmonella* spp. in raw milk and the dairy farm environment (feces, soil, feed, water) in one reaction. Brain heart infusion (BHI) broth was selected for the enrichment step to increase the density of the target bacteria by using an incubation of 4 h before multiplex RT-PCR. The results showed that the detection limit of the multiplex real-time assay was approximately 10^2^ CFU/mL for pure cultures and artificially contaminated milk without enrichment, while 12, 14, and 10 CFU/25 mL, respectively, for *S. aureus, L. monocytogenes*, and *Salmonella* spp. after pre-enrichment. The newly developed multiplex RT-PCR assay was applied to 46 dairy farm environmental samples and raw milk samples covering a wide variety of sample types. The results demonstrated that the multiplex RT-PCR assay coupled with the BHI enrichment broth was suitable for the simultaneous screening of *S. aureus, L. monocytogenes*, and *Salmonella* spp. in the pasture environment and in raw milk. The multiplex RT-PCR assay clearly and successfully shortened the total detection time and reduced labor compared to conventional culture-based methods for testing natural samples.

## Introduction

Waterborne and foodborne pathogens are ubiquitous in the environment. The threat to human health posed by foodborne pathogens has attracted public attention, and the incidence of illness or death caused by major known pathogens has increased worldwide (European Food Safety Authority/European Centre for Disease Control [EFSA/ECDC], 2014, 2015a,b). Infections or outbreaks caused by major foodborne pathogens can be the result of consuming contaminated foods, including beef, milk products, fresh vegetables, and contaminated water (Burgess et al., 2016). In addition, food itself is a complex system, as well as a complicated environment, which can supply the enough nutrition for the bacteria.

Milk is a very popular food around the world, supplying nutrients essential for human health. The total annual worldwide production of milk has reached 695 million tons and created a value of 117 billion in EU annual sales for the top 20 dairy companies (Papademas and Bintsis, 2010). Recently, consumption of raw unpasteurized milk has been increasingly welcomed as having enhanced nutritional qualities, taste, and benefits (Oliver et al., 2009). However, dairy farms and milk products are nutrient-rich reservoirs for microbes, especially for waterborne and foodborne pathogens; these farms and products supply a transmission pathway for bacteria when they come into contact with contaminated sources. Pathogens, including *S. aureus, L. monocytogenes*, and *Salmonella* spp., can cause mastitis and can be directly excreted into milk (Oliver et al., 2005). A survey showed that the prevalence of *S. aureus* was 25.3% amongst 51,963 raw milk samples in California (Heidinger et al., 2009). *L. monocytogenes* is also ubiquitous in farm environments and is found in contaminated animal feces, low-quality feed, unsanitary dairy farms, and even in milk (Sanaa et al., 1993; Fox et al., 2009; Latorre et al., 2010; Hunt et al., 2017). *Salmonella* spp. have been the most commonly reported foodborne pathogens isolated from bulk tanks, ranging from 0 to 11% (Oliver et al., 2009). These three pathogens can be transmitted through feces, soil, water, and feed, resulting in cross-contamination. Monitoring the dairy farm environment is a key point for controlling major pathogens and pathogenic diseases. Therefore, in order to minimize the risk of infection for consumers, the development of a rapid, accurate, and internationally accepted assay for the detection of these major pathogens in the dairy farm environment and milk products has become increasingly important for the food industry and for public health.

Classical culture-based approaches offer standardized procedures for the detection of these three foodborne pathogens (e.g., ISO standards), including culture enrichment, selective plating, and biochemical confirmation. These procedures are labor-intensive, complicated, and time-consuming (Kim et al., 2012; Garrido et al., 2013; Ma et al., 2014; Zhang et al., 2015). With the development of biotechnology, molecular assays such as PCR have increasingly gained attention for their ability to rapidly detect foodborne and waterborne microbiological pathogens in food and animal feed (Kagkli et al., 2011). Automated RT-PCR eliminates the need for an electrophoresis step after amplification (Elizaquível et al., 2011; Ma et al., 2014) and has the added advantage of reducing the risk of cross contamination. Multiplex PCR systems have also been applied to detect pathogenic bacteria. Many previous PCR and qPCR methods have been developed for the detection of *S. aureus, L. monocytogenes*, and *Salmonella* spp. and have been applied in simplex or multiplex formats (Elizaquível and Aznar, 2008; Rantsiou et al., 2008; Chua and Bhagwat, 2009; Suo et al., 2010; Garrido et al., 2013; Yang et al., 2013; Ma et al., 2014; Xiao et al., 2014). However, the limitations and reliability of PCR-based detection methods partly depend on the number of target bacterial cells, especially the copy numbers of the target molecules present in the sample (Garrido et al., 2013). Low contamination levels in food samples make the detection of target pathogens difficult and time consuming. Thus, considering the actual conditions, an enrichment step is necessary to improve the efficiency of the procedure. The important advantages of enrichment are that it increases the target pathogen concentration in the sample and physiologically resuscitates stressed or injured cells, which reduces the potential risk of sublethal bacteria.

In some countries, there is ‘zero tolerance’ for pathogenic bacteria. Due to assay detection limits, some pathogens cannot be detected, causing false negative results. Therefore, pre-enrichment is highly necessary for analyzing the environment and for tests in food industry. Real-time PCR coupled with an enrichment step has been applied for the detection of *S. aureus, L. monocytogenes*, and *Salmonella* spp. in various food samples, such as pine nuts, meats, fish, and eggs (McGuinness et al., 2009; Chen and Deutscher, 2010; Garrido et al., 2013; Wang et al., 2015). Two-step enrichment was carried out on selective media but was inconclusive regarding the necessity of selective cultivation (Taskila et al., 2012). One-step cultivation has been accepted and applied for the enrichment of foodborne and waterborne pathogens. And one-step enrichment was chosen instead of two-step enrichment for use in the multiplex PCR system to shorten the total detection time (Maks and Fu, 2013; Zheng et al., 2013). Currently, there are no investigations that have studied suitable broths to simultaneously enrich *S*. *aureus, L. monocytogenes*, and *Salmonella* spp.

In this study, a multiplex RT-PCR assay for the simultaneous detection of *S. aureus, L. monocytogenes*, and *Salmonella* spp. was developed. Our protocol is unique in that we increased the enrichment step before DNA extraction and screened media to find one suitable for these three pathogens to get the detection limit in a short time. The medium selected was evaluated for its ability to achieve high bacterial density of these three pathogens over a short period of time when cultured either individually or together. The multiplex RT-PCR assay with the enrichment step was characterized by its low limit of detection and reduced assay time compared with non-enrichment and conventional methods. To the best of our knowledge, this is the first study to report the use of multiplex RT-PCR with an enrichment step for the simultaneous detection of *S. aureus, L. monocytogenes*, and *Salmonella* spp. in milk and dairy farm samples.

## Materials and Methods

### Bacterial Strains and Culture Conditions

The bacterial strains used to test the specificity of the multiple RT-PCR in this study are listed in **Table 1**. *S. aureus* (ATCC 25923), *L. monocytogenes* (ATCC 19115) and *Salmonella typhimurium* (ATCC 14028) were obtained from the American Type Culture Collection (ATCC) and used to establish the multiplex RT-PCR. *S. aureus* strains were grown in tryptic soy broth (TSB; Beijing Land Bridge Technology Company Ltd, Beijing, China) supplemented with 10.0% NaCl and incubated at 37°C for 18 h. The other strains were cultured in TSB at 37°C for 18 to 20 h. Following the incubation, 8 mL of the enriched culture were pooled into sterile centrifuge tubes and centrifuged at 3000 rpm, 25°C for 10 min. The cell pellets were suspended in sterile peptone water (PW; 0.85% NaCl, 0.1% peptone) to obtain concentrations ranging from 10^1^ to 10^8^ CFU/mL.

**Table 1:** Specificity of the multiplex real-time PCR primers for different bacterial strains.

Bacterial stains	Source^a^	Multiplex real-time PCR results		
		*nuc*	*hlyA*	*orgC*
*Staphylococcus aureus*	ATCC 25923	+	-	-
	CICC 21648	+	-	-
	CICC 10786	+	-	-
	CMCC 41002	+	-	-
	CMCC 26003	+	-	-
	CGMCC 1.89	+	-	-
	CGMCC 1.128	+	-	-
*Listeria monocytogenes*	ATCC 19115	-	+	-
	ATCC 19114	-	+	-
	ATCC 19112	-	+	-
	ATCC 19116	-	+	-
	ATCC 19118	-	+	-
	CMCC 54002	-	+	-
*Salmonella* spp.				
*Salmonella typhimurium*	ATCC 14028	-	-	+
*Salmonella typhimurium*	CMCC 50362	-	-	+
*Salmonella enterica*	CMCC 50041	-	-	+
*Salmonella* Paratyphi B	CMCC 50094	-	-	+
Other stains				
*Campylobacter jejuni*	ATCC 33560	-	-	-
*Vibrio parahaemolyticus*	ATCC 33847	-	-	-
*Vibrio parahaemolyticus*	ATCC 17802	-	-	-
*Vibrio vulnificus*	ATCC 27562	-	-	-
*Escherichia coli* O157:H7	ATCC 43889	-	-	-
*Escherichia coli* O157:H7	CICC 10907	-	-	-
*Escherichia coli*	CMCC 44568	-	-	-
*Enterococcus faecalis*	CGMCC 1.2135	-	-	-
*Listeria welshimeri*	ATCC 43548	-	-	-
*Listeria welshimeri*	ATCC 43550	-	-	-
*Listeria innocua*	ATCC 33091	-	-	-
*Bacillus subtilis*	CGMCC 1.4255	-	-	-
*Bacillus cereus*	CMCC 70331	-	-	-
*Shigella boydii*	CGMCC 1.10618	-	-	-
*Shigella flexneri*	CGMCC 1.10599	-	-	-

*^a^ATCC, American Type Culture Collection, United States; CMCC, China Medical Culture Collection, China; CICC, China Center of Industrial Culture Collection, China; CGMCC, China General Microbiological Culture Collection Center, China.*

### Preparation of Artificially Contaminated Milk Samples

Ultra High Temperature (UHT) treated milk was purchased from a local supermarket (Shanghai, China). It was confirmed negative for *S. aureus, L. monocytogenes*, and *Salmonella* spp. by culturing in Baird-Parker agar (BP; Beijing Land Bridge Technology Company Ltd, Beijing, China), PALCAM agar (Beijing Land Bridge Technology Company Ltd, Beijing, China), and bismuth sulfite agar (BS; Beijing Land Bridge Technology Company Ltd, Beijing, China), respectively. An overnight culture of *S. aureus, L. monocytogenes*, and *Salmonella* spp. (∼10^8^ CFU/mL) was added to 1 mL of milk to final concentrations of 10^7^, 10^6^, 10^5^, 10^4^, 10^3^, and 10^2^ CFU/mL.

### Enrichment Optimization by Growth Kinetics

Five different commercial enrichment broths were evaluated to enrich samples for RT-PCR analysis: alkaline peptone water (APW; Beijing Land Bridge Technology Company Ltd, Beijing, China), BHI broth (Beijing Land Bridge Technology Company Ltd, Beijing, China), Luria-Bertani broth (LB; Beijing Land Bridge Technology Company Ltd, Beijing, China), TSB broth (Beijing Land Bridge Technology Company Ltd, Beijing, China), and PW. The effects of different enrichment broths on *S. aureus, L. monocytogenes*, and *Salmonella* spp. growth were determined from turbidity growth curves using an automatic Bioscreen C (Labsystems, Helsinki, Finland). Twenty-microlitre volumes of bacterial suspension samples at the appropriate concentrations were inoculated in a 100-well honeycomb plate containing 180 μL of the corresponding medium in each well, rendering a final viable count of 10–100 CFU, following ISO 11133-2:2003 recommendations regarding the productivity of the inocula (ISO 11133-2, 2003). In addition, 200 μL of the corresponding medium was added to three of the wells as blank controls. The honeycomb plate was placed in the Bioscreen C reader an incubated at 37°C for 48 h. The OD600 was measured at 30-min intervals, and the honeycomb plates were shaken at medium intensity for 20 s before every measurement (Xuan et al., 2017). The modified Gompertz model (Zwietering et al., 1990) was employed to fit the growth data using Origin pro 8.6 (Origin Lab Corp., Northampton, MA, United States). The equation of the modified Gompertz model is as follows:

$$Y=y_{0}+(y_{\max}-y_{0})\times \exp\{-\exp[\frac{\mu_{\max}}{y_{0}}\times (\lambda -t)+1]\}$$

There are three growth parameters in this model, namely, maximum growth rate (*U*_max_), which is an intrinsic parameter in a constant environment and describes the different multiplication rate; time to detection (TD), which is similar to the function of the lag phase time and describes the duration of time that the bacteria react to the enrichment broth and the time to reach the detection limit of turbidity; and the maximum population density (MPD), which indicates the highest level the bacteria can reach under given environmental conditions (Zheng et al., 2015).

### DNA Extraction

Bacterial DNA was extracted using a TIANamp Bacteria DNA Kit (Tiangen Biotech Beijing Co., Ltd., China) according to the manufacturer’s instructions. This method was modified according to the previous studies (Ye et al., 2013; Zhang et al., 2015), where the incubation time in lysozyme was increased to 1 h, and the incubation time in proteinase K was increased to 2 h.

### Primers and Probes Used for Multiplex Real-Time PCR

Primers and probes targeting *S. aureus, L. monocytogenes*, and *Salmonella* spp. were used as previously described with minor modifications (Day et al., 2009; Li et al., 2015; Omiccioli et al., 2009). TaqMan primers and probes were synthesized by Invitrogen Corp (Shanghai, China). The sequences of the primers and probes for the multiplex real-time PCR experiments are provided in **Table 2**.

**Table 2:** Primers and probes used in the multiplex real-time PCR.

Gene	Target bacteria	Primers/Probes	Product sizes(bp)	Reference
*nuc*	*S. aureus*	CACCTGAAACAAAGCATCCTAAA CGCTAAGCCACGTCCATATT FAM-TGGTCCTGAAGCAAGTGCATTTACGA-BHQ1	149	Li et al., 2015
*hlyA*	*L. monocytogenes*	ACTTCGGCGCAATCAGTGA TTGCAACTGCTCTTTAGTAACAGCTT ROX-TGAACCTACAAGACCTTCCAGATTTTTCGGC-BHQ1	137	Omiccioli et al., 2009
*orgC*	*Salmonella* spp.	CTTTATGATGCATTCTACCAACGACTG CCGAATCACCACTGTTAGGA VIC-CGCTTCCTGAGTCAGCCTCTTCTGAAACG- BHQ1	121	Day et al., 2009

### Multiplex Real-Time PCR

The multiplex real-time PCR reaction was carried out in a final volume of 20 μL with the following components: 2 μL of 10 × PCR Buffer (Invitrogen, United States), 1.2 μL of 50 mM MgSO_4_ (Invitrogen, United States), 0.5 μL of 10 mM dNTP mix (Invitrogen, United States), 0.2 μL of Taq DNA polymerase (5 U/mL) (Invitrogen, United States), 1 μL of template DNA per reaction tube, 0.5 μL of 10 mM primers and 0.2 μL of 10 mM probe for each strain.

A 7500 Fast real-time PCR system (Applied Biosystems, Foster City, CA, United States) was used. The cycling protocol consisted of 95°C for 10 min followed by 40 cycles of denaturation at 95°C for 15 s and annealing at 60°C for 1 min. Analysis of the results was performed using 7500 Software version 2.0.6.

### Standard Curves and Amplification Efficiency

To create RT-PCR standards and determine the amplification efficiency, pure cultures and artificially contaminated samples seeded with 10-fold diluted suspensions of *S. aureus, L. monocytogenes*, and *Salmonella* spp. were prepared as described in Sections “Bacterial Strains and Culture Conditions and Preparation of Artificially Contaminated Milk Samples,” respectively. A standard curve was obtained by using genomic DNA extracted from serial dilutions of the pure culture and the artificially contaminated samples. Each reaction was amplified in triplicate. Negative (no template) controls were included in each RT-PCR run. A direct plate counting procedure was conducted for the quantification of *S. aureus, L. monocytogenes*, and *Salmonella* spp. in pure cultures and samples. The cultures were incubated for 24 h at 37°C on BP agar for *S. aureus*, PALCAM agar for *L. monocytogenes*, and BS agar for *Salmonella* spp.

The different bacterial concentrations (log10 CFU/mL) were plotted against the corresponding *C*t-values and had a linear relationship. The amplification efficiencies (E) were determined by using the slope of the curve and applying the equation: *E* = 10^-1/slope^-1 (Kawasaki et al., 2010).

### Evaluation of the Limit of Detection (LOD) and Enrichment Time by Multiplex Real-Time PCR

The LOD was evaluated before and after the process of enrichment. To determine the LOD without the enrichment step, nine samples were prepared as follows: 3 mL of the suspensions of 10^9^ CFU/mL *S. aureus, L. monocytogenes*, and *Salmonella* were inoculated in 27 mL UHT milk to make the initial dilution. Then, 10-fold dilutions were generated to achieve the final contaminations of the three pathogens in UHT milk, which were 10^1^, 10^2^, or 10^3^ CFU/mL, as determined by plate counts. Then, DNA extraction was performed on 1 mL sample.

The enrichment optimization experiments were conducted using 10^1^ CFU values of *S. aureus, L. monocytogenes*, and *Salmonella* spp. in 25 mL of UHT milk to determine the LOD after enrichment. All samples were initially confirmed as negative for *S. aureus, L. monocytogenes*, and *Salmonella* spp. by plate count before the inoculation. Twenty-five milliliters of low contaminated samples (10^1^ CFU/25 mL) were added to 225 mL of selected enrichment broth and grown at 37°C. The cell growth was monitored by collecting 1 mL of the sample for DNA extraction at hourly intervals during a 5-h incubation, which was described in Section “DNA Extraction.”

Multiplex RT-PCR was conducted as described in Section “Multiplex Real-Time PCR,” and the results were gathered from both the non-enrichment tube and enrichment broth and statistically compared to evaluate the effect of the culturing time on the enrichment step.

### Detection of *S. aureus, L. monocytogenes*, and *Salmonella* spp. in Raw Milk and Dairy Farm Samples

A total of 46 milk and environmental samples were tested in this study, including fifteen normal raw milk samples, seven mastitis milk samples, six soil samples, six feed samples, six fecal samples, and six water samples from dairy farms in three areas of China. The sampling time for all samples did not exceed 5 h, and the samples were kept in the cold before extraction. The samples were analyzed by multiplex RT-PCR with enrichment and using standard culture methods for *S. aureus, L. monocytogenes*, and *Salmonella* spp. (ISO 11290-02, 1998; ISO 6579, 2002).

We added 25 mL/g of samples into 225 mL of selected enrichment broths weighed into a sterile plastic bag and enriched for a certain number of hours at 37°C in preparation for DNA extraction. The enrichment time was determined by the results of the previous step [see Evaluation of the Limit of Detection (LOD) and Enrichment Time by Multiplex Real-Time PCR]. One milliliter of supernatant was transferred to a new tube and centrifuged at 12,000 rpm for 10 min for DNA extraction, and then multiplex RT-PCR was carried out. For the standard culture method, 25 mL/g samples were added to the enrichment broths weighed into a sterile plastic bag and incubation for 24 h at 37°C. After enrichment, 100 μL was transferred to selective agar (BP agar for *S. aureus*, PALCAM agar for *L. monocytogenes* and BS agar for *Salmonella* spp.) and incubation for 48 h at 37°C. The typical colonies were picked to make the biochemical confirmation where the presumptive *S. aureus, L. monocytogenes*, and *Salmonella* spp. colonies were validated using the API STAPH test (BioMérieux), the API LISTERIA test (BioMérieux) and API 20E test (BioMérieux), respectively.

### Statistical Analysis

Statistical analyses were performed using SPSS 19.0 software (SPSS Inc., Chicago, IL, United States).

## Results

### Specificity of Primers and Probes for Multiplex Real-Time PCR

The specificity and sensitivity of the designed primers and probes (**Table 1**) were tested individually and in combination using various bacterial strains available for this study. As shown in **Table 1**, none of the target bacteria showed a negative signal; all produced a specific band corresponding to the amplicons. By contrast, non-target bacteria did not produce an amplified signal. Such results indicated that the multiplex RT-PCR was specific to *S. aureus, L. monocytogenes*, and *Salmonella* spp. and did not amplify products from other species.

### Enrichment Optimization Based on Growth Kinetics and Growth Parameters

**FIGURE 1:**
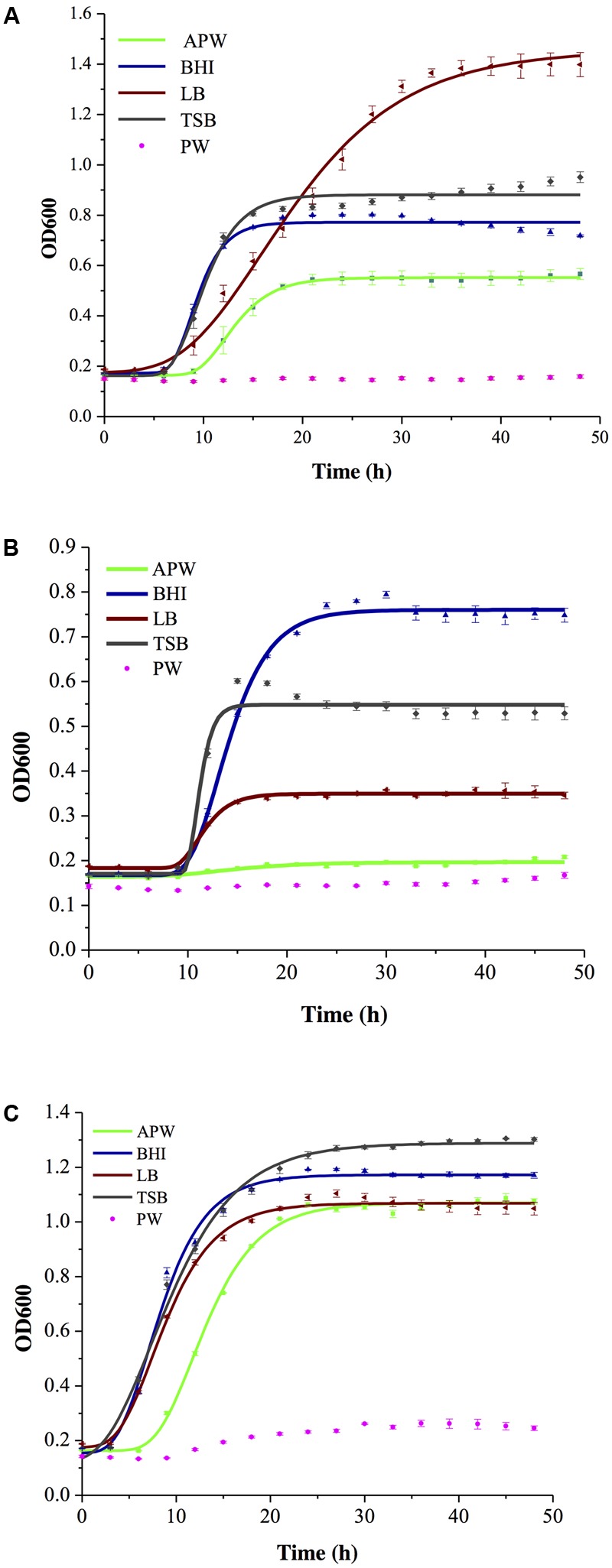
Growth curves of *S. aureus* **(A)**, *L. monocytogenes* **(B)**, and *Salmonella* spp. **(C)** in five enrichment broths.

The average growth curves of *S. aureus, L. monocytogenes*, and *Salmonella* spp. in the five enrichment broths were obtained by fitting to the modified Gomperz model and were shown in **Figures 1A–C**. The coefficients of determination (*R*^2^-values) for the fitted growth curves (APW, BHI, LB, and TSB) were greater than 0.98 (data not shown). These three pathogens grew vigorously in APW, BHI, LB, and TSB broths. However, as shown in **Figures 1A–C**, the growth of *S. aureus, L. monocytogenes*, and *Salmonella* spp. in PW was insufficient to develop a full growth curve; therefore, growth could not be fitted to the model. PW could maintain the survival of the bacteria but could not provide the necessary nutrients for bacterial growth.

The growth parameters of these three pathogens in the five enrichment broths were also calculated using the modified Gompertz model, and they are shown in **Table 3**. *S. aureus* and *Salmonella* spp. had the highest growth rate in BHI broth. This increased growth rate allowed these target pathogens to grow rapidly and reach a detectable level, shortening the detection time. The highest MPDs achieved by *S. aureus, L. monocytogenes*, and *Salmonella* spp. were in LB, BHI, TSB, respectively, and the lowest were in APW, APW and LB, respectively. The aim of the enrichment step was to facilitate the bacterial density reaching the same concentrations of the limit of detection as without enrichment in a short time. Therefore, *U*_max_ is the most important factor in the selection of media. The results obtained indicate that BHI broth is the best of these five commercial broths for the enrichment of *S. aureus, L. monocytogenes*, and *Salmonella* spp. before multiplex real-time PCR.

**Table 3:** The growth parameters of three pathogens in five enrichment broths.

		APW	BHI	LB	TSB	PW
*S. aureus*	*U*_max_	0.062 ± 0.004^*c^	0.093 ± 0.022^a^	0.022 ± 0.004^d^	0.089 ± 0.048^b^	NF
	TD	9.445 ± 0.221^b^	6.830 ± 0.541^d^	7.904 ± 0.925^c^	14.41 ± 0.366^a^	NF
	MPD	0.552 ± 0.004^c^	0.773 ± 0.016^b^	1.455 ± 0.027^a^	0.880 ± 0.611^b^	NF
*L. monocytogenes*	*U*_max_	0.032 ± 0.015^d^	0.058 ± 0.006^c^	0.100 ± 0.013^b^	0.195 ± 0.024^a^	NF
	TD	7.617 ± 0.350^a^	10.189 ± 0.344^c^	9.113 ± 0.387^b^	9.988 ± 0.153^c^	NF
	MPD	0.195 ± 0.148^d^	0.760 ± 0.010^a^	0.349 ± 0.003^c^	0.547 ± 0.005^b^	NF
*Salmonella* spp.	*U*_max_	0.042 ± 0.004^b^	0.046 ± 0.010^a^	0.048 ± 0.007^a^	0.024 ± 0.009^c^	NF
	TD	7.850 ± 0.303^a^	3.697 ± 0.495^b^	3.799 ± 0.400^b^	2.297 ± 0.768^c^	NF
	MPD	1.069 ± 0.128^c^	1.163 ± 0.027^b^	1.068 ± 0.019^c^	1.287 ± 0.044^a^	NF

*U_max_, maximum rate of growth; TD, time to detection, the time to reach the detection limit of turbidity and is measured in hours; MPD, the highest level the bacteria can reach under given environmental conditions; NF, none fit. Values are the mean of triplicate measurements ± standard deviation; values with different lowercase letters in the same row showed a significant difference at *P* < 0.05.*

### Standard Curve and Amplification Efficiency

**FIGURE 2:**
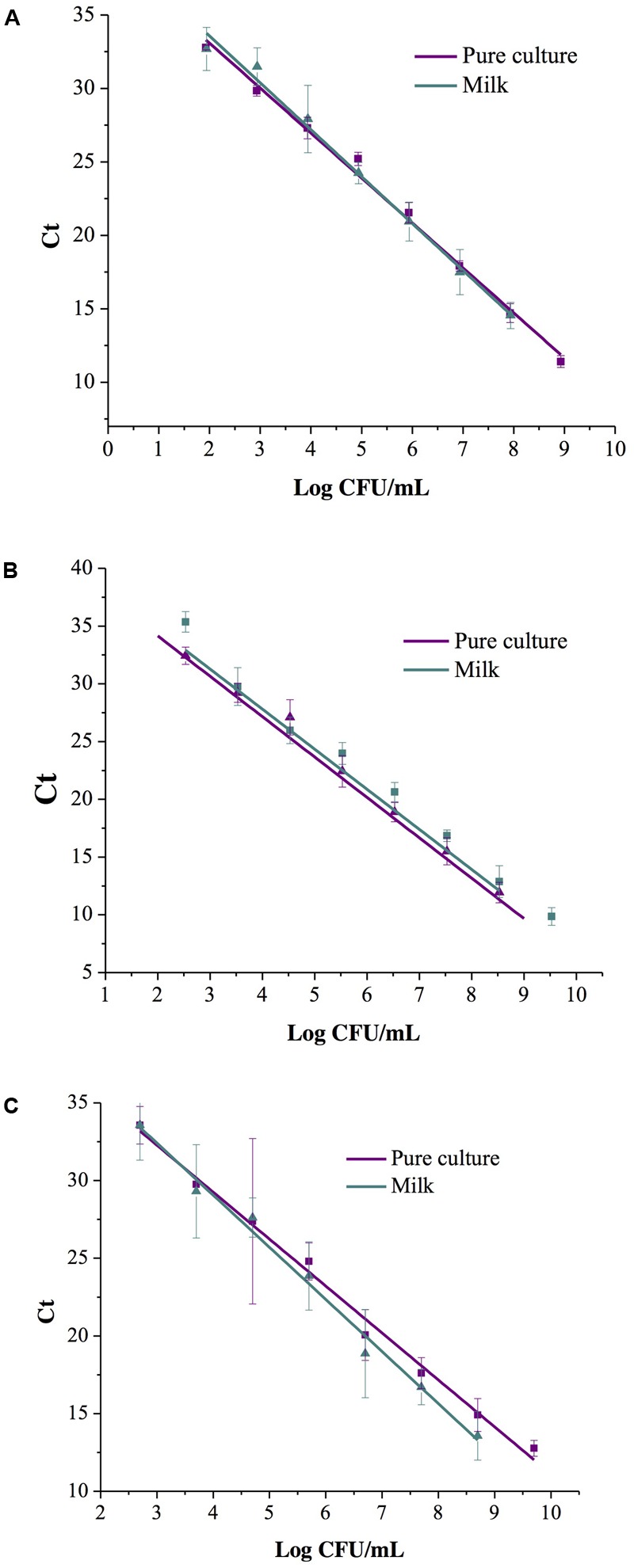
Standard curves of the multiplex real-time PCR for three pathogens of *S. aureus* **(A)**, *L. monocytogenes* **(B)**, and *Salmonella* spp. **(C)** in pure culture and artificially contaminated milk.

Three replicates were analyzed for the efficiency evaluation of the multiplex real-time PCR method in pure culture and in contaminated milk. In our standard curves, the correlations (*R*^2^) for pure culture and contaminated milk were greater than 0.99, indicating high linearity (**Figure 2**). The slopes of the linear regression curves for the pure cultures were -3.06 for *S. aureus*, -3.50 for *L. monocytogenes* and -3.02 for *Salmonella* spp. The amplification efficiencies for *S. aureus, L. monocytogenes*, and *Salmonella* spp. were 112, 95, and 114%, respectively, in pure culture. In artificially contaminated milk, the slopes were -3.19 for *S. aureus*, -3.47 for *L. monocytogenes*, and -3.35 for *Salmonella* spp., and the amplification efficiencies for *S. aureus, L. monocytogenes*, and *Salmonella* spp. were 106, 94, and 98%, respectively.

### Evaluation of the LOD in Artificially Contaminated Milk Samples Using Multiplex Real-Time PCR with and without Enrichment

For evaluation of the LOD in artificially contaminated milk samples using multiplex RT-PCR with and without enrichment, two groups of 18 samples were analyzed following the procedure described in Section “Evaluation of the Limit of Detection (LOD) and Enrichment Time by Multiplex Real-Time PCR.” The LOD results are listed in **Table 4**. The first group of samples, which had no enrichment, gave a LOD for *S. aureus, L. monocytogenes*, and *Salmonella* spp. of 10^2^ CFU/mL for each pathogen, with *C*t-values between 27 and 31. For the second group, when incubated for 1, 2, or 3 h, the *C*t-values were higher than 35 and showed the negative results. However, when incubated for 4 h of the enrichment step, the multiplex RT-PCR system could detect these three pathogens successfully, and gave a LOD of 12 CFU in 25 mL for *S. aureus*, 14 CFU in 25 mL for *L. monocytogenes*, and 10 CFU in 25 mL for *Salmonella* spp., with *C*t-values lower than 30.

**Table 4:** Evaluation of the LOD for simultaneous detection of *S. aureus, L. monocytogenes*, and *Salmonella* spp. with and without enrichment step.

	LOD		
	*S. aureus*	*L. monocytogenes*	*Salmonella* spp.
Without enrichment	10^2^ CFU/mL	10^2^ CFU/mL	10^2^ CFU/mL
With enrichment	12 CFU/25mL	14 CFU/25mL	10 CFU/25mL

### Detection of Three Major Bacteria in Samples Based on Multiplex Real-Time PCR with and without Enrichment and Using Standard Culture Methods

In total, 46 samples collected from three areas of China were analyzed for *S. aureus, L. monocytogenes*, and *Salmonella* spp. infection using both the multiplex real-time PCR method (with enrichment) and the culture method. The results obtained from the multiplex real-time PCR method and standard culture method are listed in **Table 5**. In the case of the 46 environmental samples of *S. aureus*, 11 (23.9%) were positive by the multiplex real-time PCR method and were found in raw milk, mastitis milk, and animal feed, however, 10 (21.7%) were positive based on standard culture method. From the 46 samples analyzed for *L. monocytogenes*, 4 (8.7%) were positive by the both multiplex real-time PCR method and standard culture method and were found in raw milk, mastitis milk, feces, and animal feed. There were no *Salmonella* spp. positive samples among the 46 natural samples by using both RT-PCR method and standard culture method. Compared to the RT-PCR method, the culture method was time-consuming and labor-intensive, taking 1 week for the whole process. In addition, when screened on selective medium, many species of bacteria appeared on one plate, causing difficulty in picking single colonies because of the non-specific selectivity of the medium.

**Table 5:** Results obtained for milk and dairy farm environment samples with multiplex real-time PCR (with enrichment step) and standard culture method.

Sample type	P/T^a^		*S. aureus* (PCR/culture)^b^			*L. monocytogenes* (PCR/culture)			*Salmonella* spp. (PCR/culture)		
	PCR	Culture	+/+	+/-	-/+	+/+	+/-	-/+	+/+	+/-	-/+
Raw milk	5/15	5/15	4	0	0	1	0	0	0	0	0
Mastitis milk	4/7	4/7	3	0	0	1	0	0	0	0	0
feces	3/6	3/6	2	0	0	1	0	0	0	0	0
Soil	0/6	0/6	0	0	0	0	0	0	0	0	0
Feed	3/6	2/6	1	1	0	1	0	0	0	0	0
Water	0/6	0/6	0	0	0	0	0	0	0	0	0

*^a^P/T, Positive sample number/Total sample number. ^b^+/+, positive for both real-time PCR and standard culture method; +/-, positive for real-time PCR method and negative for standard culture method; -/+, negative for real-time PCR method and positive for standard culture method.*

## Discussion

Detection of multiple pathogens in the same system is currently needed for the dairy industry to reduce the cost of detecting each pathogen (Kim and Bhunia, 2008). Multiplex PCR can be used for the detection of multiple pathogens in various industries, including the simultaneous detection of *Cronobacter sakazakii, S. aureus*, and *Bacillus cereus*, as well as the simultaneous detection of *Vibrio parahaemolyticus, L. monocytogenes*, and *Salmonella* spp. (Wolffs et al., 2007; Yang et al., 2013; Li et al., 2015; Zhang et al., 2015; Alves et al., 2016; Li F. et al., 2016). This was the first study to investigate the simultaneous growth of *S. aureus, L. monocytogenes*, and *Salmonella* spp. in commercial enrichment broth. We developed a rapid and efficient multiplex RT-PCR assay for the simultaneous detection of *S. aureus, L. monocytogenes*, and *Salmonella* spp. in milk and dairy farm environment with a pre-enrichment step. Our multiplex real-time PCR showed excellent specificity for the simultaneous detection of three target pathogens when placing these three probes and primers in one single reaction system, and they showed strong strain specificity and exclusivity (**Table 1**). This specificity was confirmed by the detection of seventeen target strains and fifteen non-target strains. The results for the pure cultures and artificially contaminated milk showed highly efficient detection (**Figure 2**).

In the present study, the limit of detection of this multiplex real-time PCR was 10^2^ CFU/mL in artificially contaminated milk without enrichment. Previous studies have identified the LOD of these three pathogens in food samples without enrichment. Forghani et al. (2016) developed a multiplex RT-PCR for the simultaneous detection of *Bacillus cereus, L. monocytogenes*, and *S. aureus* in milk, rice, and lettuce with an LOD value of 3.7 × 10^3^ CFU/g. Xiao et al. (2014) obtained an LOD value of 3.5 × 10^2^ CFU/mL for *Salmonella* and *L. monocytogenes* and of 3.5 × 10^3^ CFU/mL for *S. aureus* in food by high-resolution melting real-time PCR. Compared to previous studies, the LOD values obtained from our method are one order of magnitude lower. The reasons for the lower LOD we got were because the specific probes and suitable reaction conditions used in our reaction system. However, there is some uncertainty about the limit of detection due to the lack of pre-enrichment before multiplex RT-PCR.

Pathogens in environmental samples and the food matrix may exist in very low numbers and may sometimes be accompanied by competitor organisms. This results in difficulty in the detection of pathogens using either molecular-based or culture-based methods without a pre-enrichment step (Baylis et al., 2000; Margot et al., 2015). Most of the bacteria in marketed foods are usually in an injured/stressed state, and some are in a viable but non-culturable (VBNC) state due to heat, radiation, or low temperature treatments. *S. aureus, L. monocytogenes*, and *Salmonella* spp. have been demonstrated to possess the ability to enter the VBNC state when under extreme conditions (low temperature, tap water) (Ramamurthy et al., 2014; Li J. et al., 2016). At present, most of the multiplex or simplex PCR studies do not involved in the pre-enrichment step and there are no clear conclusions about the suitable broths and enrichment time for the pre-enrichment step. In this study, one of the major advantages was to enrich the three target bacteria in one broth and to obtain a lower LOD. The aim of the pre-enrichment step was to resuscitate the stressed target bacteria in a natural sample. BHI has been used to resuscitate VBNC *L. monocytogenes* (Lindbäck et al., 2010), which helped the cells recover from stressful conditions. In addition, BHI broth has been evaluated as an enrichment medium for *Salmonella* spp. in sprout-related studies (Tu et al., 2003; Kisluk and Yaron, 2012). As shown in **Table 3**, the growth rate of *S. aureus* and *Salmonella* spp. in BHI broth was faster than in the other enrichment broths. Therefore, BHI was the most suitable broth for the enrichment of the three targeted pathogens in raw milk and in dairy farm environmental samples using a short enrichment time. The results for artificially contaminated milk samples showed that the detection limits for all three targeted pathogens were 12 CFU/25 mL for *S. aureus*, 14 CFU/25 mL for *L. monocytogenes*, and 10 CFU/25 mL for *Salmonella* spp. (**Table 4**).

Previous studies have reported selective broths for enrichment of one or two among *S. aureus, L. monocytogenes*, and *Salmonella* spp. using one or two steps (Dailey et al., 2014; Khueankhancharoen et al., 2016). The use of mTA10 broth with 100 mM buffer and secondary enrichment to detect *Salmonella* spp. and *Listeria monocytogenes* has been reported by Garrido et al. (2013). The enrichment process was relatively complicated and time consuming. Rappaport-Vassiliadis soy broth (RVS) has also been used as enrichment media for the selection of *Salmonella* spp. (Van Schothorst and Renaud, 1985). Selective medium is more specific than non-selective medium to prevent the growth of non-target bacteria and to reduce the impact of competitor microorganisms on the growth of target bacteria (Kim and Bhunia, 2008; Taskila et al., 2012). Clearly, selective broth is more suitable for one or two species of bacteria when the targeted cells are at high concentration (Chen et al., 1993). However, we are committed to the simultaneous detection of three bacteria, and therefore, a single selective medium is not suitable for the pre-enrichment process. The development of enrichment medium for the simultaneous isolation of several pathogenic bacteria in one system is a major trend. The use of non-selective medium and the control of the enrichment time can effectively increase the concentration of the target bacteria. Compared to the other non-selective broths, *S. aureus, L. monocytogenes*, and *Salmonella* spp. had the highest growth rate in BHI broth. We controlled for the enrichment time, 4 h, which avoided the overgrowth of the background flora.

Microbial transfer in farm soil, animal feed, feces, water, and herds may result in the contamination of milk products. Doyle et al. (2016) demonstrated that the dairy farm environment in which the herds were kept in was the primary driver of the composition of the milk microbiota. While it is clear that our multiplex RT-PCR method could accelerate the analysis of milk and dairy farm environmental samples (soil, water, feed, and feces) for *S. aureus, L. monocytogenes*, and *Salmonella* spp. contamination, its success is linked to the ability of these three target bacteria to compete with resident micro-flora. In this study, a total of 46 samples of raw milk, mastitis milk, feces, soil, animal feed, and water from three areas of China were detected using multiplex RT-PCR with 4-h incubation in BHI broth. In these natural samples, 15 samples yielded positive results (**Table 5**). *S. aureus* was detected in four samples of raw milk, three samples of mastitis milk, two samples of feces and two samples of feed. *L. monocytogenes* was detected in one sample each of raw milk, mastitis milk, feces and feed. Lastly, *Salmonella* spp. could not be detected in milk or dairy farm environmental samples. Our results suggest that the sample contents and the microflora background do not affect the PCR amplification. We recommend 4 h of pre-enrichment time in BHI broth before the multiplex real-time PCR for simultaneous detection of *S. aureus, L. monocytogenes*, and *Salmonella* spp. in milk and dairy farm environmental samples.

A drawback of the present study is that the method could not accurately quantify the viable bacteria in the environment and food samples. As a result, the DNA of some dead bacteria was also included in the information of the total bacterial DNA. However, when the samples were enriched, the numbers of viable bacteria were increased in the sample, which can reduce the risk of false positive results due to the DNA of dead bacteria. Although propidium monoazide (PMA) coupled with multiplex RT-PCR is a useful tool for the quantification of viable bacteria (Bae and Wuertz, 2012; Yang et al., 2013), it still a drawback to the limit of detection. Li et al. (2015) combined DNA and PMA to quantify the viable *Legionella pneumophila, S. typhimurium*, and *S. aureus* in tap and river water, and this method could detect 10^1^ CFU/mL viable bacteria. Therefore, the pre-enrichment step combined with the multiplex PCR method is more suitable for the detection of pathogenic bacteria in the food environment and in complicated food matrixes, especially for the detection of bacteria in a few numbers.

## Conclusion

This study presents a tool for the simultaneous detection of *S. aureus, L. monocytogenes*, and *Salmonella* spp. The pasture environment and animal feed can be assessed to reduce the risk of microbial cross-contamination in the aquaculture environment, especially the bacteria in the aquaculture chain, including soil, water, feed, and feces. Multiplex RT-PCR coupled with the pre-enrichment step can be used to recognize the potential infection risk of sublethal and VBNC bacteria by allowing them to recover from stressful conditions.

## Author Contributions

TD and YS drafted the manuscript. All authors listed, have edited the manuscript, and made substantial and direct contribution to the work. All authors gave approval for publication of the manuscript.

## Conflict of Interest Statement

The authors declare that the research was conducted in the absence of any commercial or financial relationships that could be construed as a potential conflict of interest.
